# Electrochemical Immunoassay for Tumor Marker CA19-9 Detection Based on Self-Assembled Monolayer

**DOI:** 10.3390/molecules27144578

**Published:** 2022-07-18

**Authors:** Zheng Wei, Xiaoping Cai, Weifeng Cui, Junping Zhang

**Affiliations:** 1 Cancer Research Institute, Henan Academy Institute of Chinese Medicine, Zhengzhou 450000, China; questwz@163.com (Z.W.); ccxpfan@126.com (X.C.); 2 School of Basic Medicine Sciences, Henan University of Chinese Medicine, Zhengzhou 450002, China

**Keywords:** self-assembled membrane, gold electrode, electrochemical immunosensor, CA19-9, gastric cancer

## Abstract

A CA19-9 electrochemical immunosensor was constructed using a hybrid self-assembled membrane modified with a gold electrode and applied to detect real samples. Hybrid self-assembled membranes were selected for electrode modification and used to detect antigens. First, the pretreated working electrodes were placed in a 3-mercaptopropionic acid (MPA)/β-mercaptoethanol (ME) mixture for 24 h for self-assembly. The electrodes were then placed in an EDC/NHS mixture for 1 h. Layer modification was performed by stepwise dropwise addition of CA19-9 antibody, BSA, and antigen. Differential pulse voltammetry was used to characterize this immunosensor preparation process. The assembled electrochemical immunosensor enables linear detection in the concentration range of 0.05–500 U/mL of CA19-9, and the detection limit was calculated as 0.01 U/mL. The results of the specificity measurement test showed that the signal change of the interfering substance was much lower than the response value of the detected antigen, indicating that the sensor has good specificity and strong anti-interference ability. The repeatability test results showed that the relative standard deviations were less than 5%, showing good accuracy and precision. The CA19-9 electrochemical immunosensor was used for the actual sample detection, and the experimental results of the standard serum addition method showed that the RSD values of the test concentrations were all less than 10%. The recoveries were 102.4–115.0%, indicating that the assay has high precision, good accuracy, and high potential application value.

## 1. Introduction

The specific monoclonal antibody 1116NS199 to the glycoantigen CA19-9 was isolated in 1981 from a colorectal adenocarcinoma cell line and an immune murine hybridoma product with a molecular weight of 210 kD. The CA19-9 antigenic determinant cluster is a tumor cell ganglioside called sialylated type II lactate fucose. It is present in the epithelium of the pancreas, biliary tract, stomach, intestine, endometrium, and salivary glands [1,2,3]. CA19-9 is an antigenic substance associated with adenocarcinoma produced by adenocarcinoma cells and drains into the blood circulation via the thoracic duct, thereby causing elevated CA19-9 levels in peripheral blood. The critical reference value for CA19-9 in normal human serum is 37 U/mL [4]. If this indicator is significantly elevated, it can indicate a gastrointestinal tumor. The increase in CA19-9 is especially significant in gastric cancer with liver metastasis and pancreatic metastasis, which is better than other tumor markers [5,6,7,8]. Clinical methods such as chemiluminescence (CL) and electrochromic luminescence (ECL) are mainly used to detect CA19-9 [9,10,11,12], but there are shortcomings such as expensive reagents and cumbersome operation. Therefore, it has become a challenge to develop new immunoassay technologies that reduce the cost of testing and accelerate the speed of testing for mass screening.

The label-free electrochemical immunosensor is a newly developed novel detection method [13,14,15,16]. This type of immunosensor does not require the complex labeling of antigens or antibodies. It detects the target by recognizing the change of signal response during the reaction of biological macromolecules and has the advantages of high sensitivity, simple operation, fast response, easy miniaturization, and low price [17,18,19,20,21]. Self-assembled membranes (SAMs) are a class of highly ordered, structurally defined ultrathin organic membranes formed by the spontaneous assembly of reactive molecules on a solid surface through non-covalent bonding interactions under equilibrium conditions [22,23,24]. We can artificially select the properties of the self-assembled membrane material and the functional groups on the electrode surface to obtain the interface with specific physical and chemical properties needed for our experiments. SAMs have the advantage of biomimicry and biocompatibility, so they are widely used to prepare chemical and biochemical sensitive components. This work presents a SAM constructed using β-mercaptoethanol (ME) and 3-mercaptopropionic acid (MPA) for electrochemical immunoassay of CA19-9.

## 2. Results and Discussion

**Figure 1:**
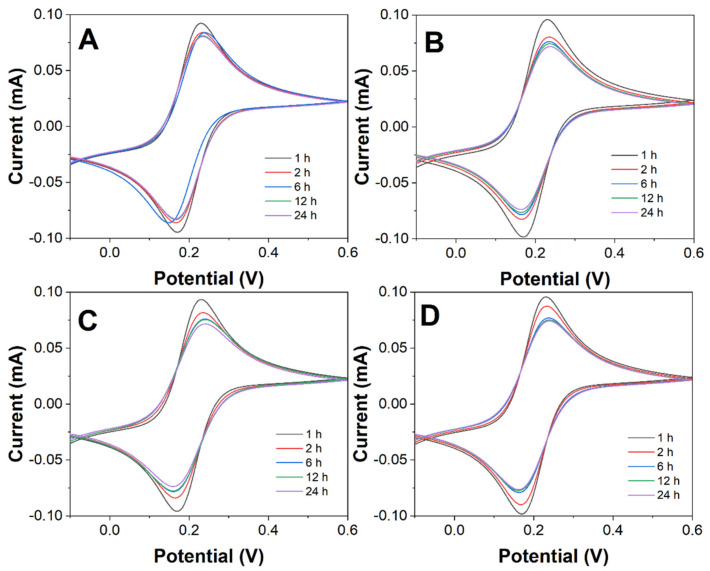
CV curves of MPA/ME/Au with MPA volume percentage of (**A**) 100%, (**B**) 50%, (**C**) 25%, and (**D**) 10%. Scan rate: 50 mV/s.

Figure 1 shows the CV curves of MPA/ME hybrid self-assembled membranes in an electrolyte for real-time in situ characterization. The variation of CV curves with time for different volume percentages of MPA was recorded. The peak current value of the CV decreased gradually with the extension of the assembly time, and the peak current value gradually stabilized at 24 h. Compared with the self-assembled monolayer film, the redox peak current decreases more slowly as the MPA volume percentage decreases for the same time for the hybrid self-assembled film. This is due to the intrinsic properties of the MPA film adsorbed on the electrode surface. If the content of MPA in the composite film formed on the electrode surface is reduced, it has less influence on the electrical conductivity of the electrode [25,26].

**Figure 2:**
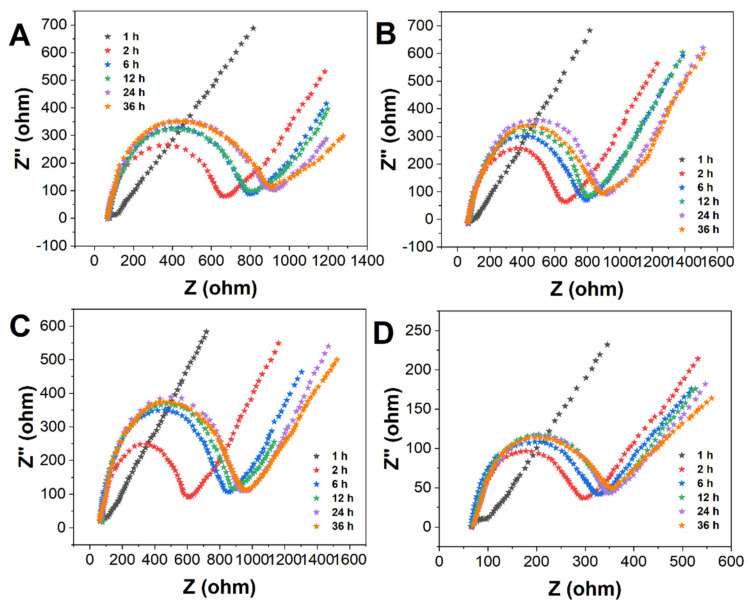
EIS plots of MPA/ME/Au with MPA volume percentage of (**A**) 100%, (**B**) 50%, (**C**) 25%, and (**D**) 10%.

Figure 2 shows the Nyquist plot of real-time in situ characterization of the MPA/ME hybrid self-assembled membrane in the electrolyte. It records the variation of EIS with time for different volume percentages of MPA, and its semicircle diameter indicates the magnitude of the impedance value. The impedance value of the MPA/ME hybrid self-assembled membrane gradually increased with the extension of the assembly time and reached saturation adsorption at 24 h. As the volume percentage of MPA decreases, the impedance value becomes smaller when the MPA/ME hybrid self-assembled membrane reaches saturation adsorption [27], and the results are consistent with the CV characterization results.

**Figure 3:**
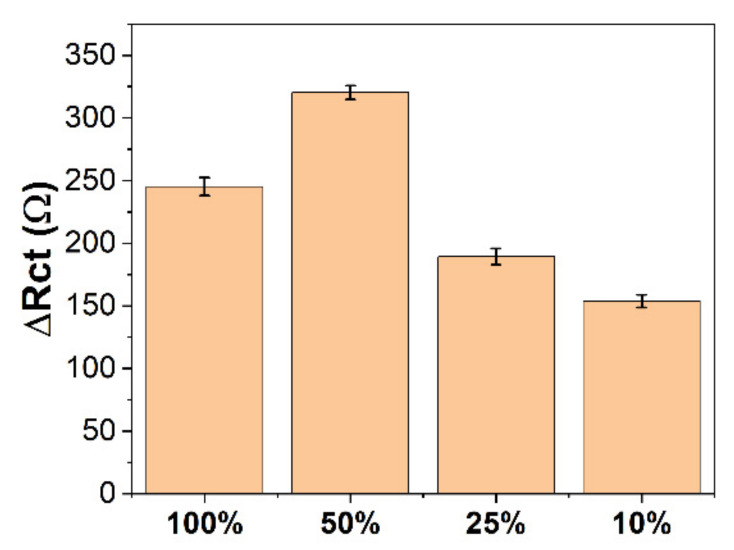
ΔRct of MPA/ME/Au with MPA volume percentage of 100%, 50%, 25%, and 10% for detecting CA19-9.

The EIS plots obtained for different volume fractions with different assembly time conditions were simulated in the circuit to obtain the ΔRct values for different assembly time (t) conditions, as shown in Table 1 and Figure 3. From Table 1, it can be found that the volume fraction of MPA has a significant effect on the self-assembly rate of MPA on the gold surface. Only the assembly of MPA monolayers shows the smallest ΔRct when the assembly time is less than 2 h. However, with the increase in assembly time, the ΔRct containing 10% MPA has the smallest. This may be because the electrode assembly containing 10% MPA does not cover the entire electrode area. The highest ΔRct at 24 h was obtained at 25% MPA, indicating that 25% MPA and 75% ME could result in a high coverage of monomolecular mixed film on the electrode surface. In either case, the electrodes were almost saturated after 24 h of assembly. Further increases in assembly time do not change much of the electrode’s performance. It can be seen that MPA/ME has the most significant change in impedance value at 25% volume percentage, which represents the most sensitive immunosensor.

**Figure 4:**
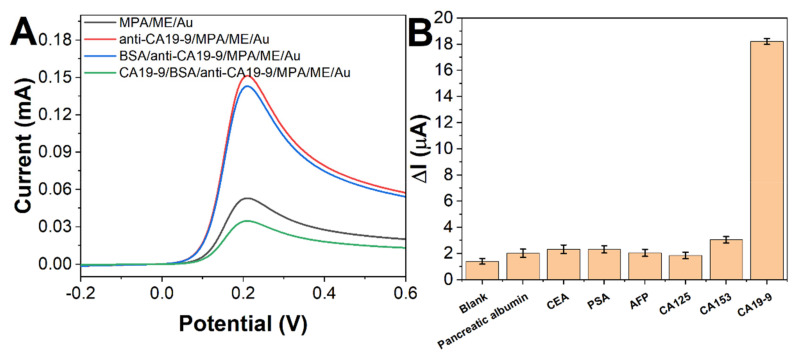
(**A**) DPV curves of MPA/ME/Au, anti-CA19-9/MPA/ME/Au, BSA/anti-CA19-9/MPA/ME/Au, and CA19-9/BSA/anti-CA19-9/MPA/ME/Au in 5 mM [Fe(CN)_6_]^3−/4−^. Scan rate: 20 mV/s. (**B**) Electrochemical detection of 5 ng/mL pancreatic albumin, CEA, PSA, AFP, and 5 U/mL CA125, CA153, CA19-9.

The electrochemical characterization of the different modification steps in the fabrication of the immunosensor to detect CA19-9 is shown in Figure 4A. Figure 4A shows the DPV plots of the sensor for different modification steps in ferricyanide electro pair solutions, showing the oxidation peaks of potassium ferricyanide changing on the electrode surface [28,29]. As can be seen from the figure, the oxidation current of potassium ferricyanide increased after immobilization of the CA19-9 antibody on MPA/ME/Au. The antibody-modified electrode slightly reduced the oxidation current of potassium ferricyanide (anti-CA19-9/MPA/ME/Au) closed with BSA. After the reaction of the modified electrode with CA19-9 and immobilization of CA19-9, the oxidation current of potassium ferricyanide was significantly reduced relative to that of the antibody-modified electrode. The DPV results confirm that the oxidation current response changes gradually with the gradual modification of the electrode [30].

DPV tested the specificity of the sensor under the same experimental conditions to detect the response of the prepared sensor to common interferers in serum. As shown in Figure 4B, the value of the change in the sensor current response signal for similar concentrations of protein markers is significantly smaller than the change in the current signal for CA19-9. Thus, the prepared sensor was able to differentiate the signals of CA19-9 and other interferents significantly.

**Figure 5:**
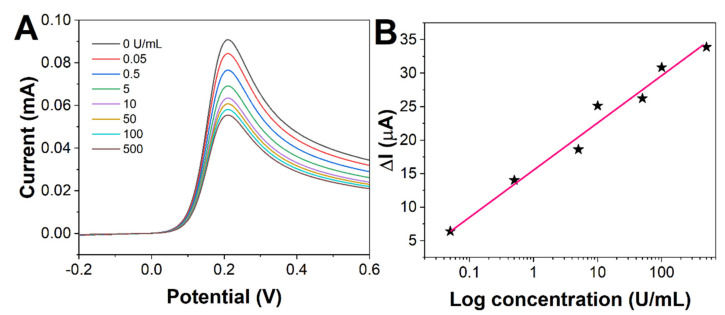
(**A**) DPV curves of BSA/anti-CA19-9/MPA/ME/Au towards 0, 0.05, 0.5, 5, 10, 50, 100, and 500 U/mL. (**B**) Plots of ΔI against the logarithm of concentration.

We measured different concentrations of CA19-9 with BSA/anti-CA19-9/MPA/ME/Au. As shown in Figure 5A, the oxidation peak current of DPV gradually decreased with the increase in the CA19-9 concentration. The value of the current variation showed a good linear relationship with the logarithmic value of CA19-9 concentration in the range of 0.05 to 500 U/mL (Figure 5B). The linear regression equation was: ΔI (μA) = 15.81 + 7.122logC (U/mL). The limit of detection can be calculated as 0.01 U/mL based on a signal-to-noise ratio of 3. Meanwhile, we compared the performance of the immunosensor prepared in this experiment with those reported in the literature. As can be seen from Table 2, the immunosensor prepared in this experiment for detecting CA19-9 was very competitive.

**Table 2:** Comparison of BSA/anti-CA19-9/MPA/ME/Au with previous published electrochemical immunosensors for CA19-9 detection.

Sensor	Linear Range (U/mL)	Limit of Detection (U/mL)	Reference
Anti-CA19-9/3D-ordered macroporous magnetic Au film	0.05–15.65	0.01	[31]
Microfluidic chip	10.75–172	10.75	[32]
Anti-CA19-9/AuNPs/poly(thionine)-SDS Nanocomposites	6.5–520	0.26	[33]
Antibody–AuNP–G-quadruplex/hemin	0.025–1	0.016	[34]
AuPt nanocalliandras	0.05–50	0.03	[35]
BSA/anti-CA19-9/MPA/ME/Au	0.05–500	0.01	This work

The reproducibility of the immunosensor is an important indicator of the practical performance of the immunosensor [36]. To evaluate the reproducibility of the immunosensor, the immunosensor prepared from the same batch of five electrodes was assayed against the same concentration of CA19-9, and the relative standard deviation (RSD) was obtained. The current response of the five individual sensors to the same concentration (5 U/mU) of CA19-9 was measured, and the RSD was 2.5%, indicating that the immunosensor has good reproducibility. We investigated the reserve stability of the immunosensor by placing the sensor in a refrigerator (4 °C) and testing the sensor in the same test solution every 7 days. The current response of the sensor decreased by 3.1% after 7 days. After three weeks, the current response values of the immunosensor were 85% of the initial values, indicating that the reserve stability of the immunosensor was good.

We used a spiked recovery assay to determine CA19-9 in serum. This was performed by adding l U/mL, 3 U/mL, and 5 U/mL of different concentrations of CA19-9 antigen solution to the serum samples, and the results are shown in Table 3. As can be seen from Table 3, the relative standard deviations were between 2.09% and 3.54%, indicating the excellent precision of the method. The recoveries ranged from 98.7% to 102.4%, indicating the high accuracy of the method. The results indicate that the test method can be initially applied to the clinical determination of CA19-9 levels in serum samples.

## 3. Experimental

### 3.1. Reagents and Instruments

CA 19-9 and anti-CA19-9 (Ab) were purchased from Shanghai Leadtek Biotechnology Co. (Shanghai, China). Potassium ferricyanide, disodium hydrogen phosphate, sodium dihydrogen phosphate, dipotassium hydrogen phosphate, and potassium dihydrogen phosphate were purchased from Tianjin Kaitong Chemical Reagent Co. (Tianjin, China). 1-ethyl-(3-dimethylaminopropyl)carbodiimide hydrochloride (EDC) was purchased from Shanghai Aladdin Biochemical Technology Co. (Shanghai, China). N-hydroxysuccinimide (NHS), β-mercaptoethanol (ME), and 3-mercaptopropionic acid (MPA) were purchased from Shanghai Maclean Biochemical Technology Co. (Shanghai, China). All other reagents were analytical grade and used without further purification.

An electrochemical analyzer (CHI660C, Shanghai Chenhua Instruments Co., Ltd., Shanghai, China) was used for all electrochemical experiments. The three-electrode system is a working electrode of gold (3 mm diameter), a counter electrode of platinum wire, and a reference electrode of Ag/AgCl.

### 3.2. Preparation of Electrochemical Immunoassay

The gold electrode was soaked in the freshly prepared piranha solution (3:1 mixture of sulfuric acid and 30% hydrogen peroxide) for 10 min and the electrode was removed and rinsed with deionized water. The electrodes were polished to a mirror finish with alumina powder of 0.3 μm and 0.05 μm. The electrodes were cleaned and scanned (cyclic voltammetry, CV) in 0.5 M sulfuric acid from −0.2 to 1.5 V (scan rate: 100 mV/s) until stable electrochemical behavior was obtained.

The pretreated gold electrodes were assembled in a mixture of 5 mL of 5 mM MPA and 5 mM ME mixture solution (100%, 50%, 25%, and 10% of MPA volume) at room temperature for 1–24 h to form a hybrid self-assembled membrane MPA/ME (denoted as MPA/ME/Au). The CV and impedance profiles of the hybrid self-assembled membrane electrodes were measured by CV, and electrochemical impedance spectroscopy (EIS) was used to characterize their film formation efficiency.

The gold electrode with the assembled self-assembled membrane was removed, cleaned, and placed in 5 mL of EDC/NHS mixed solution (0.40 M EDC and 0.10 M NHS) for 1 h and then washed with PBS. Twenty microliters of 1 mg/mL of anti-CA19-9 was added dropwise on the electrode surface and assembled overnight at 4 °C. The unbound or poorly bound antibodies on the electrode surface were washed with water and dried. The electrode was immersed in 1% BSA solution, and then the electrode surface was washed with PBS.

### 3.3. Electrochemical Detection of CA19-9

The electrodes were characterized in situ using EIS, CV (scan rate: 50 mV/s) and differential pulse voltammetry (DPV) in a mixed solution (10 mL) of 10 mM [Fe(CN)_6_]^3−/4−^ + 0.1 mol/L KCl in PBS with pH = 7.4. The perturbation amplitude for EIS detection is 5 mV, using frequencies from 0.1 Hz to 100,000 Hz. The conventional Randles equivalent circuit is used for simulation. Twenty microliters of CA19-9 solution was added dropwise on the surface of the final prepared working electrode, incubated for 30 min at 37 °C, washed with PBS, and dried. The assay of CA19-9 in serum using an electrochemical immunosensor was performed by the standard addition method. The operation was consistent except that the spiked serum replaced the CA19-9 standard.

## 4. Conclusions

Immobilizing antibodies by single-component self-assembled membranes results in considerable spatial site resistance due to the high density of functional groups at the reaction interface, leading to a decrease in the efficiency of immobilized antibodies and tending to cause larger ones non-specific adsorption. Therefore, for effective immobilization of the CA19-9 antibody, MPA/ME was used to form a hybrid self-assembled membrane on the gold electrode. In this study, we successfully constructed an electrochemical immunosensor based on the hybrid self-assembled membrane to detect CA19-9 with a linear range of 0.05–500 U/mL with a detection limit of 0.01 U/mL.

## Figures and Tables

**Table 1:** ΔRct changes at different volume fractions of MPA with time.

MPA (%)	1 h	2 h	6 h	12 h	24 h
100	73.31	150.23	221.64	338.20	584.30
50	261.10	635.21	740.22	766.20	878.30
25	242.40	488.50	719.30	805.22	1120.30
10	204.14	228.51	244.63	251.20	262.28

**Table 3:** The results of analysis of serum samples.

Sample	Add (U/mL)	Found (U/mL)	RSD (%)	Recovery (%)
1	1.00	0.97, 1.03, 1.07, 1.15, 0.95	8.05	115.0
2	3.00	3.05, 2.98, 2.91, 3.09, 3.03	6.94	103.0
3	5.00	5.08, 5.01, 4.89, 4.93, 5.12	9.71	102.4

## Data Availability

The data presented in this study are available on request from the corresponding author.

## Sample Availability

Samples of the compounds are not available from the authors.
